# ALK receptor activation, ligands and therapeutic targeting in glioblastoma and in other cancers

**DOI:** 10.3389/fonc.2012.00192

**Published:** 2012-12-19

**Authors:** Anton Wellstein

**Affiliations:** Lombardi Cancer Center, Georgetown UniversityWashington, DC, USA

**Keywords:** anaplastic lymphoma kinase, growth factor, pleiotrophin, midkine, signal transduction

## Abstract

The intracellular anaplastic lymphoma kinase (ALK) fragment shows striking homology with members of the insulin receptor family and was initially identified as an oncogenic fusion protein resulting from a translocation in lymphoma and more recently in a range of cancers. The full-length ALK transmembrane receptor of ~220 kDa was identified based on this initial work. This tyrosine kinase receptor and its ligands, the growth factors pleiotrophin (PTN) and midkine (MK) are highly expressed during development of the nervous system and other organs. Each of these genes has been implicated in malignant progression of different tumor types and shown to alter phenotypes as well as signal transduction in cultured normal and tumor cells. Beyond its role in cancer, the ALK receptor pathway is thought to contribute to nervous system development, function, and repair, as well as metabolic homeostasis and the maintenance of tissue regeneration. ALK receptor activity in cancer can be up-regulated by amplification, overexpression, ligand binding, mutations in the intracellular domain of the receptor and by activity of the receptor tyrosine phosphatase PTPRz. Here we discuss the evidence for ligand control of ALK activity as well as the potential prognostic and therapeutic implications from gene expression and functional studies. An analysis of 18 published gene expression data sets from different cancers shows that overexpression of ALK, its smaller homolog LTK (leukocyte tyrosine kinase) and the ligands PTN and MK in cancer tissues from patients correlate significantly with worse course and outcome of the disease. This observation together with preclinical functional studies suggests that this pathway could be a valid therapeutic target for which complementary targeting strategies with small molecule kinase inhibitors as well as antibodies to ligands or the receptors may be used.

Receptor kinase activities are controlled by expression levels as well as the binding of their cognate ligands (Schlessinger and Ullrich, 1992; Schlessinger, 2000). Furthermore, mutations can inhibit or enhance constitutive receptor activity as well as ligand-dependent signaling. The anaplastic lymphoma kinase (ALK) tyrosine kinase receptor fits into this general model though the kinase was discovered as part of an oncogenic fusion protein of the intracellular portion of ALK with nucleophosmin (NPM) induced by a t(2,5) translocation (Morris et al., 1994). A series of other fusion partners with the intracellular ALK portion were discovered thereafter (Duyster et al., 2001), most recently as a transforming oncogene in a small subset of lung cancers. These fusion proteins have gathered much interest as a therapeutic target for small molecule kinase inhibitors and are reviewed elsewhere in this issue. The intracellular kinase portion of ALK show ~50% homology and overlapping signaling paths with the insulin receptor family, including the signaling through the adaptor molecule insulin receptor substrate-1 (IRS-1), SHC, and Grb2 (see **Figure 1**; Ueno et al., 1996; Stoica et al., 2001; Crockett et al., 2004; Kuo et al., 2007). The full-length ALK was identified as a transmembrane receptor and its highest expression was found in the developing central and peripheral nervous system (Iwahara et al., 1997; Morris et al., 1997). Expression of the full-length protein was also found in cultured normal cells and in cancer cell lines (Stoica et al., 2001). It is noteworthy that the extracellular domain (ECD) of ALK shares significant homology over a ~40% stretch with the leukocyte tyrosine kinase (LTK) and is otherwise a unique protein (Duyster et al., 2001; see **Figure 1**).

**FIGURE 1:**
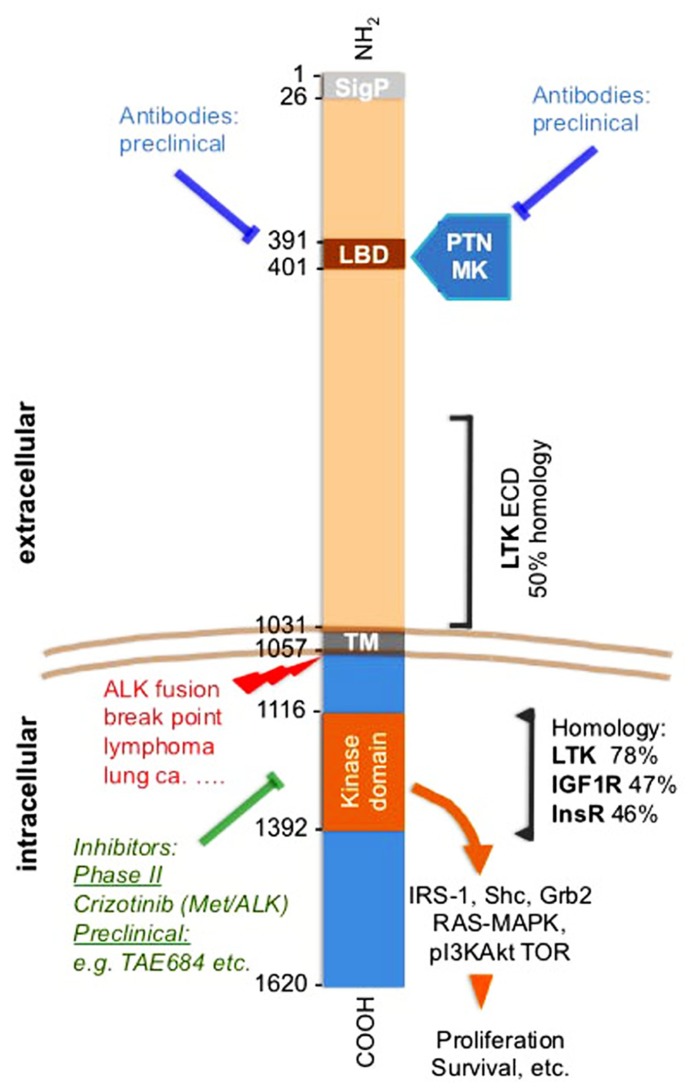
**Anaplastic lymphoma kinase (ALK) receptor domains, pathways, inhibitors, and homologies**. Amino acid positions flanking different domains in ALK are shown. Small molecule kinase inhibitors and antibodies are indicated as is the ALK translocation breakpoint. The amino acid sequence overlap and homology of the ECD of LTK is shown. Also, the homology with the closest related kinases is indicated for the kinase domain. Mutations discussed in the text and also in (Wellstein and Toretsky, 2011). The LBD was identified by phage display of a brain cDNA library against immobilized PTN (Stoica et al., 2001). The LBD is within an MAM domain that is frequently found in the ECDs of a diverse family of transmembrane proteins (Prosite data base PDOC 00604). SigP, signal peptide; LBD, ligand binding domain; ECD, extracellular domain; ICD, intracellular domain; TM, transmembrane domain; PTN, pleiotrophin; MK, midkine; LTK, leukocyte tyrosine kinase; IGF1R, insulin-like growth factor 1 receptor; InsR, insulin receptor.

## LIGANDS OF THE ALK RECEPTOR

**FIGURE 2:**
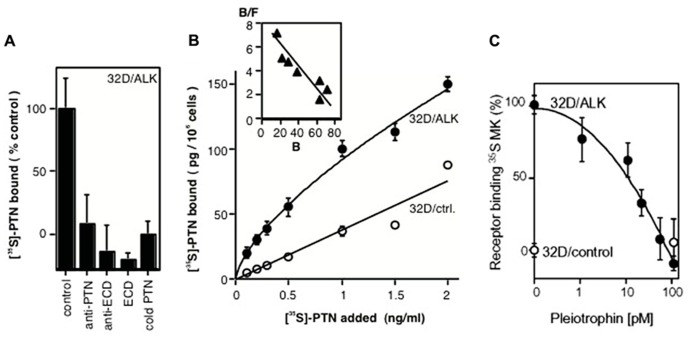
**Binding of PTN and MK to the ALK receptor in intact cells**. **(A)** Competition for ALK binding of ^35^S-PTN in 32D/ALK cells. Competitors were: anti-PTN antibody, anti-ECD antibody, ECD protein, unlabeled PTN. **(B)** Titration of equilibrium binding of ^35^S-PTN to 32D/ALK and to 32D/control cells. A Scatchard analysis of specific binding is shown in the inset. Modified from Stoica et al. (2001). **(C)** Competition of PTN for ALK receptor binding of ^35^S-MK. Binding of ^35^S-MK to 32D/ALK (filled symbols) and 32D/control cells (open symbols) was competed by different concentrations of PTN. Modified from Stoica et al. (2002).

Our studies of growth factors and their signal transduction connected the ALK receptor with the growth factor pleiotrophin (PTN): We had purified and characterized PTN from supernatants of human breast cancer cells (Fang et al., 1992; Wellstein et al., 1992) and defined a signaling pathway of this protein (Souttou et al., 1997). PTN is highly expressed in the nervous system in addition to its high expression in some cancers (Li et al., 1990; Fang et al., 1992). From this we reasoned, that the likely receptor would also be expressed in brain tissues. To identify a receptor for PTN, we thus used a phage library that displayed human brain cDNAs as fusion proteins on the phage surface. Purified PTN was immobilized as a bait protein, and the phage library was panned against this bait. From this unbiased screen we identified a phage insert sequence that matched with a region in the ECD of the ALK receptor that was only cloned a year earlier (Iwahara et al., 1997; Morris et al., 1997). This amino acid stretch in ALK thus defined the ligand binding domain (LBD) in the receptor (**Figure 1**; Stoica et al., 2001). A recombinant ECD of ALK incubated with immobilized PTN validated the findings with the phage. Also, a titration curve of PTN against the immobilized ALK ECD showed saturable binding with an EC_50_ of PTN around 1 ng/ml. In a complementary, unbiased screen, mass spectrometry analysis of cancer cell supernatants identified PTN as the major ligand for the ALK ECD that had been immobilized on SELDI mass spectrometry chips (Stoica et al., 2001). Finally, a series of receptor binding studies in intact cells completed the analysis: equilibrium binding of ^35^S-PTN to ALK that was expressed in 32D cells was inhibited by an antibody to PTN as well as an antibody to the LBD, an excess of the ECD protein and by an excess of unlabeled PTN protein (**Figures 2A**,**B**). This data support a specific ligand-receptor interaction. The interaction was further quantitated by receptor binding studies in ALK transfected 32D cells with ^35^S-PTN. The K_D_ of 30 pM (= 0.5 ng/ml) derived from a Scatchard analysis (**Figure 2B**) matched well with the EC_50_ of purified PTN in earlier growth assays and connected protein interactions with functionality (Wellstein et al., 1992). Also, a series of phosphorylation studies with different intracellular substrates showed that signal transduction initiated as early as 1 min after addition of the PTN ligand (Stoica et al., 2001). In parallel experiments the PTN homolog midkine (MK) was studied for its signal transduction and receptor interaction with ALK. We observed a somewhat lower affinity of MK for the ALK receptor (~100 pM). MK binding was competed by unlabeled PTN in a concentration range that matches the affinity of PTN (**Figure 2C**). Furthermore, signal transduction of MK as well as stimulation of colony formation induced by exogenously added MK were inhibited by monoclonal antibodies raised to the ALK LBD (Stoica et al., 2002). From these data we conclude that MK and PTN are activating ligands for the ALK receptor.

## THE PHYSIOLOGIC ROLE OF THE PTN/MK–ALK AXIS

A well established paradigm is the close relationship of pathways that control malignant progression and pathways that contribute to tissue repair and tissue maintenance. Furthermore, molecules that participate in physiologic development of a particular tissue are likely to contribute significantly to its repair after injury as well as to its malignant progression. With the discovery that new neurons and glia are produced throughout life from undifferentiated, pluripotent precursor cells, it became obvious that such stem cells may also participate in the initiation and progression of nervous system tumors (Holland, 2001; Vescovi et al., 2006). PTN, MK, and ALK are highly expressed in the nervous system during development. A recent report connected MK and ALK signaling to sympathetic neuron growth during development and aberrant signaling to neuroblastoma predisposition (Reiff et al., 2011). The increased expression of PTN, MK, and ALK in more advanced stages of cancers of glial origin was shown earlier (see below), and matches with a partial reversion of tumor cells to a precursor phenotype that was operational during development.

Recent studies also show a significant role of the PTN–ALK interaction during neuronal injury (Mi et al., 2007) and thus connect this growth factor-receptor pathway to adult tissue repair. Interestingly, germ line inactivation of ALK, PTN, or MK did not impact on overall embryo survival or cause gross pathologies. However, a more detailed analysis revealed subtle alterations in animal behavior, memory, and fertility in knockout animals (Muramatsu et al., 2006; Zou et al., 2006; Bilsland et al., 2007; reviewed in Li and Morris, 2007). Also, more recent studies in MK knockout mice showed that MK counteracts the deposition of amyloid plaques suggesting a role in repair (Muramatsu et al., 2011). PTN knockout mice revealed altered amphetamine-seeking behavior (Gramage et al., 2010) and this is complemented by a role of ALK in behavioral responses to ethanol (Lasek et al., 2011). If one extrapolates from these studies in rodents to potential side effects of targeted therapies, the subtle phenotypic outcomes of gene deletions suggests that therapeutic targeting may not cause severe side effects. It is a matter of conjecture to predict whether higher nervous system functions may be impacted by such treatments.

## ALK, LTK PTN, AND MK EXPRESSION IN CANCER

### EXPRESSION IN THE STROMA OF CANCER TISSUES

**FIGURE 3:**
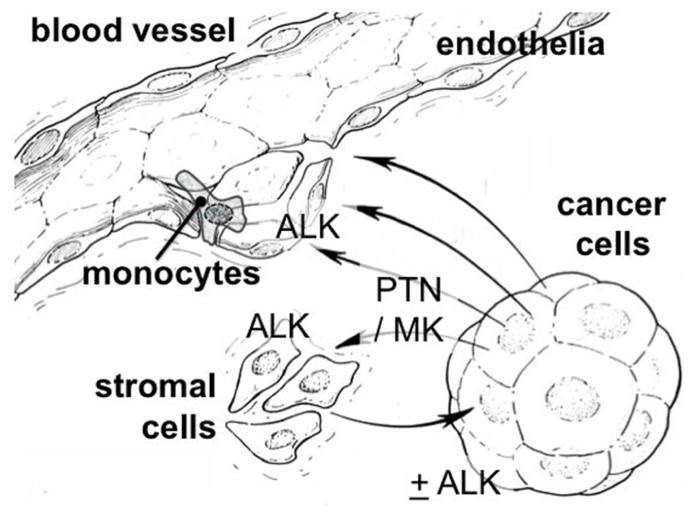
**Cross-talk between stromal and cancer cells via the PTN/MK–ALK pathway**. PTN and MK are heparin-binding proteins released from cancer or stromal cells. They can bind at nanomolar affinity to glycosaminoglycans (GAGs) such as the heparan sulfate side chains of proteoglycan (HSPG) as well as chondroitin sulfate (CS; Deepa et al., 2002). CS is proposed as a co-receptor for MK (Muramatsu, 2010) as are other GAGs (Li et al., 2010). Also *N*-syndecan could function as such a co-receptor (Raulo et al., 1994). Both ligands and receptor can be found up-regulated in the stroma of cancer as well as in cancer epithelia (see text).

The cross-talk between cancer cells and stromal cells is a crucial element of malignant progression and is required for cancer cell migration, invasion into the vasculature, the recruitment of inflammatory cells, and the establishment and expansion of organ metastases. The scheme in **Figure 3** illustrates this. To assess expression of the PTN–ALK pathway genes in stromal tissues, we analyzed several data sets from microdissection and transcriptome analysis of cancer and normal stroma. Finak et al. (2008) reported an analysis of stromal gene expression in a breast tissue set. Analysis of their data shows a significant up-regulation of ALK (*p* = 5 × 10^–8^), LTK (*p* = 3 × 10^–4^), and PTN (*p* = 0.002) in the stroma of breast cancers relative to the stroma of normal breast tissues. An analysis of the stroma of pancreatic duct adenocarcinoma showed only LTK (*p* = 5 × 10^–4^) and PTN (*p* = 0.002) significantly up-regulated relative to normal pancreatic stromal tissues (Buchholz et al., 2005). No significant changes were seen in a further analysis of breast tissues (Karnoub et al., 2007). These data sets were accessed through the Oncomine data base (Rhodes et al., 2004, 2007).

### BRAIN TUMORS

The normal developmental expression pattern of the ligands PTN and MK in different tissues overlap to some extent with the ALK receptor. The overall highest levels are found in the central and peripheral nervous system during mid to late gestation (Li et al., 1990; Iwahara et al., 1997). In normal adult tissues, however, only limited expression of the full-length ALK receptor, PTN or MK was seen but overexpression of these genes was reported in a number of human cancers including brain tumors (Schulte and Wellstein, 1997; Mentlein and Held-Feindt, 2002; Powers et al., 2002; Peria et al., 2007). More recently, we evaluated the expression of ALK and PTN mRNA in normal brain and 34 glial tumor tissues using *in situ* hybridization of serial sections of surgical specimen. Overall, only the most aggressive, high grade tumors, i.e., GBM (glioblastoma multiforme) and anaplastic oligodendroglioma showed an increased expression of PTN and ALK mRNA relative to normal brain tissues, relative to adjacent brain tissues and relative to low grade tumors (*p* < 0.01). Also there was a direct correlation between ALK and PTN mRNA expression (*p* < 0.001) that was also visible when superimposing serial sections of the tissues that had been hybridized with different probes (Stylianou et al., 2009).

An analysis of published gene expression data sets corroborates this finding (**Table 1**; Rhodes et al., 2004, 2007): GBM express significantly higher levels of PTN and ALK than normal brain tissues and astrocytoma or oligodendroglioma. Interestingly, the phosphatase PTPRz that is thought to interact with the ALK pathway (see below) is also significantly up-regulated in all of these cancer specimen relative to normal brain.

**Table 1:** Brain tumor gene expression.

	Expression levels relative to normal brain (*n* = 23; data from Sun et al., 2006)		
	AC	ODG	GBM
*n*	26	50	77
PTN	0.0004	<0.0001	<0.0001
MK	<0.0001	0.0001	<0.0001
ALK	NS	NS	0.013
PTPRz	<0.0001	<0.0001	<0.0001

*mRNA expression of PTN, MK, ALK, PTPRz in normal brain versus brain tumors from the Oncomine data base were used for the analysis. The number of patients in each group and the *p*-value for the comparisons of expression levels of the respective gene are indicated. NS, not significant, *p* > 0.05. AC, astrocytoma; ODG, oligodendroglioma; GBM, glioblastoma multiforme. Modified from Stylianou et al. (2009).*

### GENE EXPRESSION AND DISEASE OUTCOME

**FIGURE 4:**
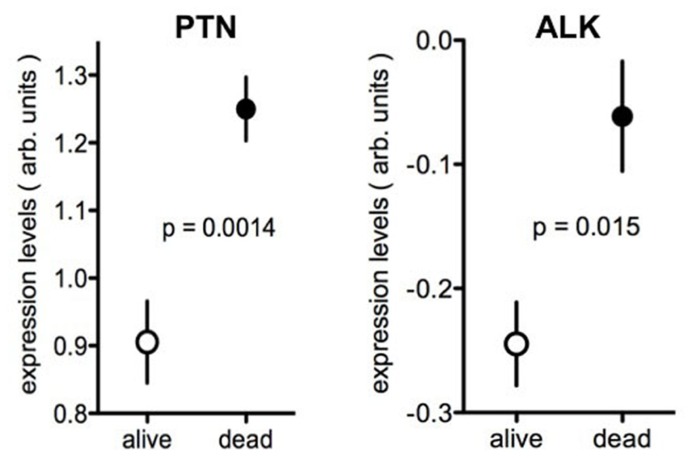
** Expression of PTN or ALK in brain tumors in patients with different survival**. Data from the Oncomine data base showing the expression levels of PTN (Phillips et al., 2006) or of ALK (Shai et al., 2003) in brain tumor samples. Tumor expression data were separated into those from patients alive or dead at 5 years (PTN) or 3 years (ALK) respectively. The data are presented as log values after normalization to the respective gene expression array and are shown as provided by Oncomine. Further studies are provided in **Tables 1 and 2**.

Comparisons of gene expression levels in clinical cancer specimen and disease outcomes can be useful for disease prognosis and in assessing whether a gene may function as driver of malignant progression: a positive correlation between higher expression levels of a given gene and poor disease outcome provides at the least a marker of poor prognosis (Mischel et al., 2003). Beyond prognosis, a correlation between gene up-regulation and poor outcome may also indicate that the gene of interest is a driver of the malignancy. HER2/erbB2 expression in breast cancer is one of the best known examples of this paradigm with high levels of expression indicating poor prognosis and at the same time favorable response to antibody therapy with anti-HER2 antibody treatment, e.g., with trastuzumab. Here we used data extracted from published genome-wide expression analyses to avoid a bias in favor of the gene sets of interest. We then assessed whether there is a relationship between tumor expression of the genes and disease outcome. To illustrate this concept, data from two brain tumor studies are depicted in **Figure 4** using published data sets (Shai et al., 2003; Phillips et al., 2006). For this, the respective gene expression arrays of surgical tumor specimen at the time of diagnosis were searched for the levels of PTN or ALK mRNA. Gene expression is provided on a log scale in arbitrary units generated after normalization by the data base provider to allow for a comparisons across platforms. PTN or ALK mRNA levels obtained from the tumor specimen were then separated into two groups, i.e., for those patients who were still alive 3 or 5 years after the initial diagnosis versus those patients who had perished by that time. The respective survival data were available for these data sets. The comparison of gene expression in these two groups shows that patients with higher expression levels of PTN or of ALK in their brain tumors had perished by the chosen time whereas patients with lower levels were still alive. Thus, higher expression of PTN or of ALK is associated with poor outcome of the disease (**Figure 4**).

In a systematic analysis of studies present in the Oncomine data base we applied this approach and found 18 independent gene expression data sets of different cancers that also provided disease outcomes, showed expression of all genes of interest and were thus suitable for the analysis. The respective outcome measures and correlation with the expression of ALK, LTK, PTN, and MK are shown in **Table 2** (Freije et al., 2004). In this series at least one of the PTN–ALK pathway genes showed a significant correlation between high expression level and poor outcome for each of the cancers represented. For several of the cancers, overexpression of more than one of the genes in this pathway were related to poor outcome. Obviously, this analysis is limited by the available gene expression data obtained from surgical tumor specimen and the necessary follow-up of the disease outcome. At the very least, this unbiased analysis supports the hypothesis that the ALK pathway may be a valid target for therapeutic interventions in cancers of different subtypes and organs including the nervous system, breast, lung, colon, ovary, prostate, and in melanoma. Limitations of the analysis are due to the available data sets and/or outcome measures.

**Table 2:** Disease outcomes relative to gene expression of ALK, LTK, PTN, and MK in clinical cancer specimen.

	Study name in Oncomine	Cancer subtype	Outcome measure	ALK	LTK	PTN	MDK
Brain tumors	Phillips brain	Astrocytoma	Dead at 3 Years Dead at 5 Years	0.045 –	– –	0.047 5.47E-05	– 0.007
	French brain	Anaplastic Oligodendroglioma	Dead at 3 Years Dead at 5 Years	– –	– –	0.033 0.034	– –
	Freije brain	Anaplastic Oligodendroglioma	Dead at 3 Years	–	–	0.019	–
	Freije brain	Glioblastoma	Dead at 1 Year	–	2.89E-04	–	–
	Pomeroy brain	Medulloblastoma	Dead at 1 Year	–	–	0.004	–
Breast cancer	Boersma breast	Ductal breast carcinoma epithelia	Dead at 5 Years	0.029	–	–	–
	Desmedt breast	Invasive ductal carcinoma	Dead at 5 Years	0.003	–	–	–
	Pawitan breast	Breast carcinoma	Dead at 3 Years Dead at 5 Years	1.30E-04 7.90E-04	– –	– –	– –
	Loi breast	Breast carcinoma	Metas. at 3 Years Metas. at 5 years	0.016 7.37E-04	0.034 –	– –	– –
	Minn breast 2	Breast carcinoma	Metas. at 1 Year Metas. at 3 Years Metas. at 5 Years	0.036 0.011 7.49E-04	– – 0.041	– – –	– – –
Colon cancer	Kurashina colon	Colon Adenocarcinoma	Dead at 1 Year Dead at 3 Years	– –	0.008 0.002	– 0.032	– 0.028
	TCGA 2 colorectal	Colon Adenocarcinoma	Dead at 1 Year	–	0.033	–	–
	Smith colorectal	Colorectal Adenocarcinoma	Dead at 5 Years	0.009	–	–	0.025
Lung cancer	Bild lung	Lung Adenocarcinoma	Dead at 1 Year Dead at 3 Years	4.35E-05 0.013	– 0.037	– –	– –
Melanoma	Xu melanoma	Melanoma	Metastasis Dead at 3 Years	0.017 –	– 0.036	– –	0.23 –
Ovarian cancer	Bild ovarian	Ovarian carcinoma	Dead at 1 Year	0.041	–	–	–
	Tothill ovarian	Ovarian serous Adenocarcinoma	Recur. at 3 Years Recur. at 5 Years	0.003 0.018	– –	– –	– –
Prostate cancer	Taylor prostate 3	Prostate carcinoma	Recur. at 1 Year Recur. at 3 Years Recur. at 5 Years	0.021 – 0.037	0.003 – –	– – –	0.014 0.035 –
All studies	Separate Studies *n* = 18	Studies (out of 18) that showed at least one outcome correlation of < 0.05		12	8	5	5

*Measures of disease outcome include overall survival (alive or dead at 1, 3, or 5 years), disease free or disease recurrence and metastasis free or occurrence of metastasis. mRNA expression levels of ALK, LTK, PTN, and MK were obtained and compared for the available disease outcomes. The respective *p*-value for these comparisons of expression levels with different disease outcomes are indicated. Details to the respective studies are available under the study names indicated in the first column (Oncomine.org; Rhodes et al., 2004, 2007). A total of 18 suitable studies were found and each of the *p*-values shown indicates a significant positive association of higher expression of the respective gene with worse outcome.*

## FUNCTIONAL PRECLINICAL STUDIES IN CANCER

We have previously shown that the ALK protein and mRNA are overexpressed in some tumors of glial origin and shown that ribozyme-mediated depletion of ALK mRNA from human U87MG glioblastoma cells resulted in apoptosis of xenograft tumors in mice (Powers et al., 2002). Different laboratories have demonstrated the significance of PTN as a growth and survival factor for different solid tumors including melanoma (Czubayko et al., 1996), pancreatic cancer (Weber et al., 2000), glioblastoma (Grzelinski et al., 2006), and multiple myeloma (Chen et al., 2007) to name a few. More recent studies show that MK contributes to glioma progression through ALK signaling and thus renders glioma cells resistant to antitumoral effects of cannabinoids (Lorente et al., 2011; reviewed in Velasco et al., 2012). Furthermore, in lung adenocarcinoma, menin and polycomb-mediated repression of PTN transcription and is proposed as an epigenetic mechanism that controls the PTN–ALK signaling pathway and hence can inhibit lung cancer progression (Gao et al., 2009).

## ALK AND CANCER CELL INVASION

**FIGURE 5:**
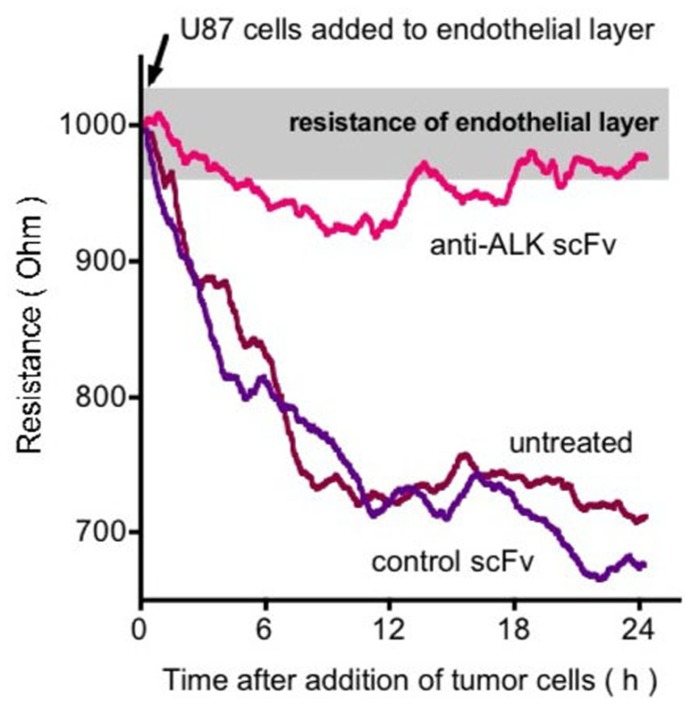
**Effect of anti-ALK antibody on U87 GBM cell invasion of an endothelial cell monolayer**. Endothelial cell monolayers were formed on electrodes and the intactness of the monolayer was monitored by electrical impedance sensing. Upon addition of the U87 cells the monolayer is disrupted and this is reflected in real-time as a decrease in electrical resistance of the monolayer. Inclusion of an anti-ALK antibody prevents this disruption (Stylianou et al., 2009).

The invasive capability of cells is a hallmark of malignancy and we thus studied the contribution of ALK signaling to tumor cell invasion of an endothelial monolayer (Stylianou et al., 2009). In a co-culture system a confluent endothelial cell monolayer was grown and then exposed to tumor cells (**Figure 5**). Electrical resistance of the monolayer was monitored as a real-time indicator of invasion (Keese et al., 2002). We used the human U87MG glioblastoma cells that express PTN with human umbilical vein endothelial cells (HUVEC) that express ALK (Stoica et al., 2001). In the invasion assay U87MG cells showed a continuous invasion that was prevented by inclusion of an anti-ALK antibody (Stylianou et al., 2009). It is noteworthy that proliferation of the tumor cells is not affected by either by depletion of ALK (Powers et al., 2002) or by blockade with an antibody. Obviously, U87MG invasion of stromal tissues can be inhibited by targeting of the PTN–ALK interaction. An ALK-dependent invasive phenotype was recently also described *in vivo* in a mouse model of pancreatic neuroendocrine carcinogenesis (Chun et al., 2010).

## ALK–PTPRz CROSS-TALK AND PTN ISOFORMS

In addition to the binding to the ALK tyrosine kinase, PTN and MK also bind to a receptor-type protein tyrosine phosphatase PTPz, PTPRz, PTPRZ1, or RPTPb/z (Maeda et al., 1996, 1999; Maeda and Noda, 1998). Work from T. F. Deuel’s laboratory showed that PTN inactivates PTPz thereby controlling the activity of tyrosine kinases (Meng et al., 2000; Perez-Pinera et al., 2006), possibly also impacting ALK signaling (Perez-Pinera et al., 2007). Furthermore, the MK–PTPz interaction was shown to induce osteoblast cell migration as well as neuronal survival (Owada et al., 1999; Qi et al., 2001; Sakaguchi et al., 2003). Interestingly, two isoforms of PTN (18 and 15 kDa) were identified more recently and appear to have distinct roles and intracellular signaling transduction pathways with respect to ALK and PTPRz (Lu et al., 2005). The PTN isoforms work at different protein concentrations with the 15 kDa isoform being more potent for ALK and the 18 kDa isoform that interacts with PTPRz being less potent. To address this distinction of pathways *in vivo*, A. Höke’s laboratory at Hopkins evaluated the contribution of PTN and potential receptors to neuronal repair (Mi et al., 2007). The laboratory had observed a striking up-regulation of PTN during spinal motor neuron denervation and sought to identify the driver pathway. Only ALK was up-regulated from a survey of potential receptors described for PTN. Antibody blockade revealed that ALK was the mediator of trophic activities of PTN in motor neurons. This is corroborated by a later study where PTN induced neurite outgrowth and signal transduction that were attenuated by anti-ALK antibodies, but not anti-PTPRz antibodies suggesting that ALK is involved in the PTN signaling on neural development (Yanagisawa et al., 2010). As mentioned above deposition of amyloid plaques (Muramatsu et al., 2011), altered amphetamine-seeking behavior (Gramage et al., 2010) and behavioral responses to ethanol (Lasek et al., 2011) were reported for MK, PTN, and ALK knockouts. An interesting connection between ligand-dependent ALK signaling, sympathetic neurogenesis, and neuroblastoma was provided recently in a study on the timing of sympathetic neurogenesis. The Rohrer laboratory showed that neurogenesis is controlled by MK–ALK and proposed that this may explain some of the ligand-mediated, ALK-dependent neuroblastoma predisposition (Reiff et al., 2011).

Overall, these reports suggest a crucial role of the PTN/MK–ALK axis in the genesis, repair and function of nervous system tissues as well as the initiation of malignancies. Cross-talk with the receptor tyrosine phospatase PTPRz appears to modulate signaling.

## GLYCOSAMINOGLYCANS AND HEPARAN SULFATE PROTEOGLYCANS

Pleiotrophin and MK are heparin-binding growth factors and their interaction with proteoglycans thus also impacts on their biologic activity (Li et al., 2007; Sugahara and Mikami, 2007), reminiscent of the impact of glycosaminoglycans (GAGs) on the signaling of other heparin-binding growth factors, most prominently FGFs (reviewed in Ornitz, 2000; Powers et al., 2000). In support of a role of GAGs for PTN–MK function, a recent study showed that a fragment of PTN (amino acids 65 to 97) binds to the GAG co-receptor of FGF2 and PTN, and inhibits the mitogenic and tumorigenic activities of both growth factors (Hamma-Kourbali et al., 2008). This may also explain the dominant-negative effect described for PTN fragments (Zhang et al., 1997; Chang et al., 2006). Also, chondroitin and dermatan sulfates (CS/DS) can bind PTN and present it to high affinity receptors (Bao et al., 2005) and more recent studies demonstrate that PTN is dependent on CS/DS binding for its neurite outgrowth activity (Li et al., 2007; Sugahara and Mikami, 2007): antibodies against PTN or ALK inhibited the neuritogenesis induced by growth factors bound to the CS/DS chains, and thus revealed the involvement of PTN and ALK. It is noteworthy that the aforementioned receptor tyrosine phosphatase PTPRz is a CS proteoglycan which may explain the tight binding of PTN or MK and its co-receptor function in growth factor signaling.

## ALK MUTATIONS AND LIGAND SIGNALING

Activating mutations in the ALK receptor were found associated with familial neuroblastoma, a fatal childhood cancer (Chen et al., 2008 George et al., 2008; Janoueix-Lerosey et al., 2008; Mossé et al., 2008). The identification of ALK mutations as potential drivers in neuroblastoma is significant for a portion of this cancer though overexpression of ALK may make a larger contribution to the malignant progression. Indeed, a recent study shows that high levels of ALK expression supersede mutations of the receptor as a determinant of poor outcome of primary neuroblastoma (Schulte et al., 2011). This could be due to the activity of endogenous ligand that will parallel the receptor level as suggested by growth inhibition even after depletion of wild-type ALK (Kishida et al., 2012).

Although ALK mutations in neuroblastoma can only account for small survival differences due to their low overall incidence, the ALK F1174 mutation (in contrast to the ALK R1275 mutation) coincides with a measurably worse outcome. This supports a possible direct impact of the F1174 mutant on signaling and malignant progression. Whilst stratification of outcomes by ALK expression levels showed significantly worse outcome with high ALK expression, multigene analyses may provide a more robust predictor and more detailed understanding of pathways that are activated. From that, one would hope to inform the use of a different drug or of drug combinations.

## CONCLUSION

Targeting of the PTN/MK–ALK signaling pathway may provide a new, mechanism-based approach to the treatment of cancers that show activation of the pathway either due to high expression levels of either these molecules or less well defined mechanisms. It is conceivable that a combination of antibody targeting of ligands or receptor plus small molecule kinase inhibitors may act synergistically and generate a better therapeutic outcome. Preclinical findings suggest only subtle side effects from targeting this pathway.

## Conflict of Interest Statement

Anton Wellstein is named as an inventor on ALK and PTN related patents of Georgetown University.
